# A rare case of perineal neuralgia treated with ultrasound‐guided nerve block combined with water separation

**DOI:** 10.1002/ibra.12138

**Published:** 2023-11-14

**Authors:** Yong Wang, Min Wang, Rui Jiang, Zhao‐Qiong Zhu, Guang‐Cai Li

**Affiliations:** ^1^ School of Anesthesiology Zunyi Medical University Zunyi Guizhou China; ^2^ Department of Anesthesiology Guizhou Aerospace Hospital Zunyi China; ^3^ Department of Anesthesiology Affiliated Hospital of Zunyi Medical University Zunyi China; ^4^ Early Clinical Research Ward of Affiliated Hospital of Zunyi Medical University Zunyi China

**Keywords:** adhesion release, neuralgia, right indirect inguinal hernia, ultrasound‐guided nerve block

## Abstract

A middle‐aged man experienced intermittent acupuncture‐like pain in the skin of the right scrotum and medial thigh 1 month after undergoing laparoscopic high ligation of the right hernial sac for a right indirect inguinal hernia in November 2020, and the pain persisted for 6 months. Under ultrasound guidance, ilioinguinal and genitofemoral nerve block and local adhesion water separation were performed with a mixture of 0.25% lidocaine (6 mL) plus prednisolone acetate (10 mg), and the same drugs and methods were repeated five times once a week. After five treatments, the visual analogue scale (VAS) score was reduced from 7 before treatment to 1. During the follow‐up period, at 3 months after the end of treatment, the VAS score remained at 0, and this score was sustained at 0 even at the 6‐month follow‐up. These outcomes indicate that ultrasound‐guided nerve block combined with water separation technology can effectively alleviate neuralgia caused by surgical adhesion, which holds important clinical significance in managing such conditions.

## Graphical abstract

A middle‐aged man presented with a right indirect inguinal hernia in November 2020 and underwent laparoscopic high ligation of the right hernial sac. Intermittent acupuncture‐like pain in the skin of the right scrotum and medial thigh occurred 1 month after surgery and lasted for 6 months. Under ultrasound guidance, ilioinguinal nerve and genitofemoral nerve block and local adhesion water separation were performed with 0.25% lidocaine 6 mL plus prednisolone acetate 10 mg mixture, and the same drugs and methods were used for five treatments (once a week). After five treatments, the visual analogue scale (VAS) score was reduced from 7 before treatment to 1. The VAS score of the follow‐up patients was 0 at 3 months after the end of treatment, and the VAS score of the follow‐up patients was still 0 at half a year. It can be seen that ultrasound‐guided nerve block combined with water separation technology can be used for neuralgia caused by surgical adhesion, which has important clinical significance.

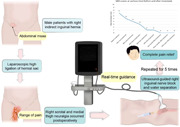

## 1 INTRODUCTION

Important structures within the inguinal canal include the spermatic cord, ilioinguinal nerve, genitofemoral nerve, and spermatic cord and arteriovenous in men.^1^ The ilioinguinal nerve originates from the first lumbar nerve, and mainly sends out to the perineum and the anteromedial aspect of the thigh to innervate the perineal area and the anteromedial aspect of the thigh and thigh for skin sensation, corresponding muscle contraction and relaxation. When the ilioinguinal nerve is stretched, compressed, or injured, it can lead to numbness of the sellar region of the human body and incontinence of fecal function. Genitofemoral neuralgia is mainly caused by compression or injury during the course of the genitofemoral nerve. The femoral branch and the femoral artery pass under the inguinal ligament and provide sensory nerve distribution to a small piece of skin on the inner thigh; after the genital branch passes through the inguinal canal, it innervates the cremaster muscle along with the spermatic cord in males and provides sensory nerves at the root of the scrotum distributed.^2^ The local soft tissue adhesion caused by the surgical operation is relatively common, and the adhered local tissue will also have acid swelling and pain. Patients undergoing laparoscopic high ligation of inguinal hernia may suffer from in the inguinal region as a result of tissue adhesion in the inguinal canal. There is usually local soft tissue pain after surgery, and the incidence rate found is 4.1%–12% in domestic literature and 0%–63% in foreign reports.^3^ The ilioinguinal nerve and genitourinary nerve belong to the structures in the inguinal canal, and the neuropathic pain caused by the entrapment of local tissue adhesion has not been reported. This rare clinical case includes soft tissue pain and neuralgia, which is a kind of complex pain. It has brought great pain to patients. If not timely handled, it will bring serious consequences, such as insomnia, anxiety, and depression.

## 2 CASE REPORT

### 2.1 Case information

**Figure 1:**
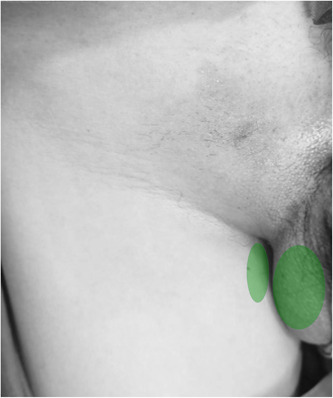
Pain range of patients: the green area indicated the pain range of the patient 6 months after surgery. [Color figure can be viewed at wileyonlinelibrary.com]

A 43‐year‐old man presented with painful symptoms in his right scrotum and medial thigh for approximately 6 months since December 2020. Earlier, on November 5, 2020, the patient visited our hospital because of a mass in the right inguinal region for more than a year and was diagnosed with an indirect inguinal hernia. Afterward, he underwent laparoscopic high ligation of the right inguinal hernia in our hospital. After the operation, the patient did not feel any obvious discomfort. However, 1 month after the operation, he developed intermittent acupuncture‐like pain in the right scrotum and medial thigh skin (Figure 1). The pain was obvious when in contact with clothes, accompanied by medium numbness with a range of pain; there was no pain in the anal region and the right groin area. After a hot compress in the right groin area and taking diclofenac sodium sustained‐release tablets, the pain was not significantly relieved. Due to the illness, there have been issues with sleep and overall mood (as indicated by a Pittsburgh Sleep Quality Index score of 16), while urination and defecation have remained normal. The patient was assessed with a self‐rating Anxiety and Depression Scale and diagnosed with no anxiety and depression. The score on the pain visual analogue scale (VAS) ranged from 6 to 7. Physical examination: There was slight tenderness in the right groin area, and touch pain was observed in the right scrotum and inner thigh. The bilateral cremaster reflex was absent, and there was superficial hypoesthesia on the medial side of the right thigh. Preliminary diagnosis: 1. Neuralgia in the perineal area (ilioinguinal nerve and/or genitofemoral nerve); 2. Inguinal canal adhesions.

### 2.2 Treatment

**Figure 2:**
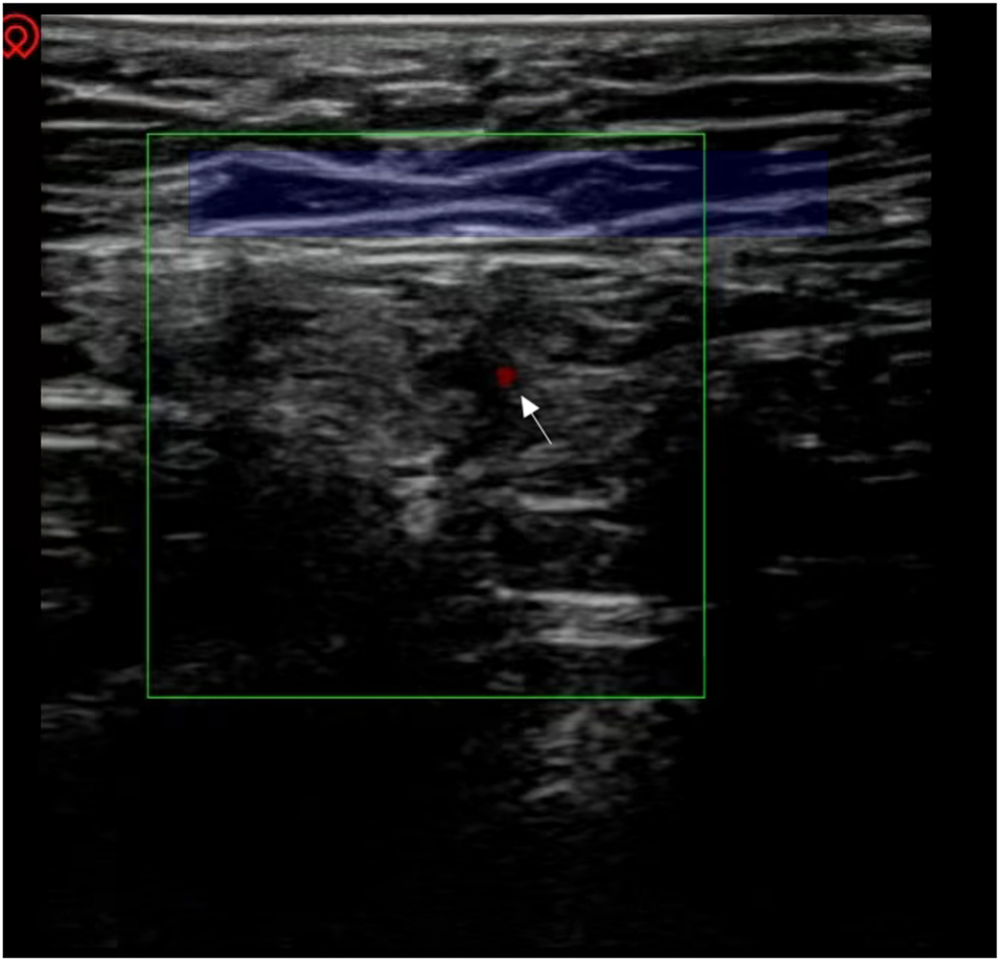
Detecting inferior epigastric artery. The green box represents the blood flow box guided by ultrasound in real‐time; the red dot, pointed out by the white arrow, indicates the inferior epigastric artery and the blue phantom area presents the inguinal canal. [Color figure can be viewed at wileyonlinelibrary.com]

**Figure 3:**
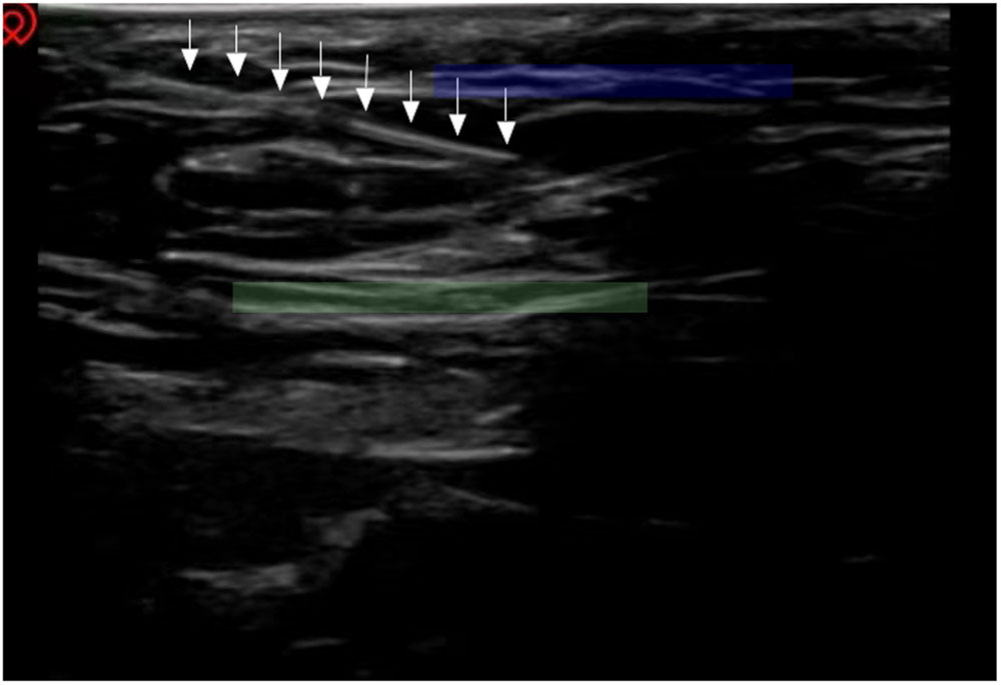
Ultrasound guided real‐time puncture. The white arrow indicates the needle track of the puncture needle. The blue area indicates the anterior wall of the inguinal canal, and the green area indicates the posterior wall of the inguinal canal.

**Figure 4:**
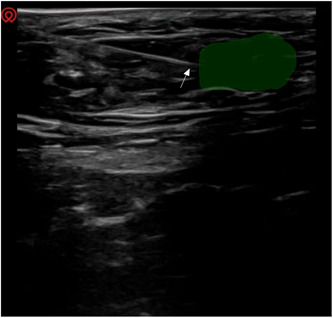
Nerve block and adhesion lysis were performed by drug injection. The white arrow indicates the needle tip, and the green area indicates the diffused drug solution. [Color figure can be viewed at wileyonlinelibrary.com]

An ultrasound‐guided block of the ilioinguinal and reproductive femoral nerves was performed at the patient's inguinal canal.^4^ First, the inferior epigastric artery was found (Figure 2). The inguinal canal was located through the inferior epigastric artery. Then, 4 mL of 0.5% lidocaine was slowly injected into the inguinal canal (Figure 3). Ten minutes later, the pain VAS score decreased from 7 to 3 and then increased to 6 after about 4 h of maintenance. Due to the insufficient pain relief provided by the nerve block, subsequently, nerve block combined with water separation in the right inguinal area was performed. Briefly, under ultrasound guidance, a mixture of 0.25% lidocaine (6 mL) and prednisolone acetate (10 mg) was used to block the ilioinguinal nerve and genitofemoral nerve combined with water separation in the groin area. The adhesion was partially separated and released (Figure 4). During the drug injection, the patient experienced soreness in the right testis and inner thigh. Five times, ultrasound‐guided ilioinguinal and genitofemoral nerve block combined with water separation was performed at 4‐week intervals. The drugs and operation procedures used were the same each time. Pain was assessed via VAS scores after each treatment, and follow‐up was performed 1 month, 3 months, and half a year after the end of treatment. Three months after treatment, the numbness in the range of pain disappeared and the patient's sleep significantly improved.

### 2.3 Results and tracked data

**Figure 5:**
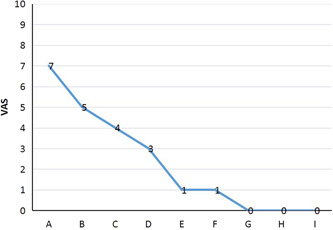
Visual analogue scale (VAS) at various times before and after treatment: (A) before treatment, (B) after one treatment, (C) after two treatments, (D) after three treatments, (E) after four treatments, (F) after five treatments, (G) 1 month after treatment, (H) 3 months after treatment, and (I) half a year after treatment. [Color figure can be viewed at wileyonlinelibrary.com]

Following blockade on the ilioinguinal and genitofemoral nerves and local adhesion separation, the patient reported pain relief after each of the five treatments. The VAS score decreased by 1–2 points after each treatment and eventually reached a score of 1–2 points. Compared to the previous treatment, the local numbness was substantially relieved; after 1 month of follow‐up, the VAS score was 0–1 with no symptoms of numbness. Three months after the treatment, the VAS score remained at 0 and remained so for half a year. After each of the five treatments, the VAS score decreased by more than 70% compared to before treatment (Figure 5), indicating a significant therapeutic effect.

## 3 DISCUSSION

Neuropathic pain can be initially diagnosed based on its symptoms and signs, manifested as spontaneous pain (discharge‐like, acupuncture‐like, burning‐like, etc.), hyperesthesia, and sensory alienation.^5^, ^6^ Neuropathic pain is a chronic pain, a pain syndrome caused by disease or damage to the somatosensory system.^7^, ^8^ It may be related to nerve damage, infection, metabolic disease, and nerve compression. One month after laparoscopic high ligation of the right indirect inguinal hernia, the skin of the right scrotum and inner thigh exhibited periodic acupuncture‐like discomfort, accompanied by heightened sensitivity at the affected area. Based on the patient's medical history and anatomy, it can be concluded that a mass caused the pain in the right groin area, which had been present for over a year. The abdominal contents and peritoneum bulge into the inguinal canal, which will secrete through the hernia sac. Following laparoscopic high ligation of the right indirect inguinal hernia, inflammatory fluid infiltrated the inguinal canal,^9^ leading to local adhesions within the internal structure of the inguinal canal due to the presence of inflammatory secretions. These adhesions involved the ilioinguinal nerve and the genitofemoral nerve, resulting in compression and stretching, ultimately causing neuropathic pain in the regions innervated by these nerves.

The patient, in our case, did not experience any noticeable paresthesia and pain within 1 month after surgery. However, after 1 month, he developed rare symptoms among outpatients, including pain, numbness, and hypoesthesia in the right scrotal and medial thigh, which lasted for 6 months. After analyzing the scar constitution and medical history of the patient, we concluded that the intermittent acupuncture‐like pain and hypoesthesia in the scrotum and inner thigh skin were closely related to the neuropathic pain caused by adhesion, compression, and traction of the structures within the inguinal canal. This was likely caused by the retraction of the intestinal canal in the dilated inguinal canal and/or by the traction of the adjacent ilioinguinal nerve and/or reproductive femoral nerve.^10^ Therefore, an ultrasound‐guided block of the ilioinguinal and reproductive femoral nerves in the inguinal region combined with local water separation and adhesion release was performed, and satisfactory results were achieved. To prevent such complications, tissue damage is minimized during surgery, and preventive measures such as using medications or devices that prevent adhesion may be considered.^11^, ^12^, ^13^

Ultrasound‐guided nerve block has been widely used in the diagnosis and treatment of neuropathic pain.^14^, ^15^ It has been reported that multiple ultrasound‐guided nerve blocks may reduce abnormal conduction of nerve impulses and reduce nerve excitability.^16^ This technique offers numerous advantages, including convenience, speed, precision, high safety, and low cost, which has gradually been incorporated into the outpatient clinic to manage chronic pain^17^, ^18^ and other emerging applications. Water separation is a surgical technique that involves pressurized fluid for tissue separation,^19^ considered a blunt technique. In clinical practice, water separation technology has a certain clinical effect in treating local adhesions that have already been formed. In this case, the patient was treated by the ultrasound‐guided block of the ilioinguinal and genitofemoral nerves at the inguinal canal, as well as water separation in the outpatient department of our hospital until complete resolution of symptoms.^20^ The results indicated that ultrasound‐guided nerve block combined with water separation can be used as one of the main treatment methods for similar cases of postoperative sequelae chronic pain. However, there are contraindications for ultrasound‐guided nerve block, including unwillingness, local or systemic infection, bleeding tendencies, severe cardiopulmonary insufficiency, and allergies to local anesthetic. In addition, it is essential to exercise caution when performing this procedure to avoid serious complications resulting from operator inexperience with ultrasound technology and unclear visualization of local anatomy. Under such circumstances, using this technology for treatment is strictly prohibited.

The treatment methods for chronic pain include physical therapy, drug therapy, rehabilitation exercise, minimally invasive therapy, open surgery, and so forth, of which minimally invasive therapy includes nerve block, radiofrequency ablation, pulsed radiofrequency, low‐temperature plasma ablation, and so forth.^21^ However, since both the ilioinguinal and genitofemoral nerves contain motor nerve fibers, radiofrequency ablation and low‐temperature plasma may lead to irreversible damage to the motor nerve fibers. Therefore, they are not suitable for use in this particular case, but a lower temperature can be considered. The treatment options for this patient include drug therapy, local physiotherapy, nerve block, peripheral nerve pulse radiofrequency therapy, and so forth. Compared with other treatment methods, nerve block combined with water separation has the advantages of rapid onset, minimal side effects, low treatment cost, and obvious curative effect. In this case, we performed periodic nerve blocks combined with water separation using the same drugs and techniques for a duration of 4 weeks. The combination of nerve block and water separation achieved satisfactory clinical outcomes in alleviating neuropathic pain caused by local adhesions that had already been formed.

In conclusion, while chronic pain in local tissues caused by surgery is relatively common, cases of neuralgia specifically caused by soft tissue adhesion and compression after surgery have rarely been reported. This particular case of perineal neuralgia caused by adhesion after inguinal surgery is infrequently encountered in clinical practice. However, through ultrasound‐guided nerve block therapy combined with water separation, we achieved a satisfactory therapeutic effect. It is recommended that ultrasound‐guided nerve block combined with water separation be used as a primary treatment method for neuropathic pain caused by local adhesion after operation as long as there are no contraindications. These findings hold certain reference significance for treating similar chronic neuralgias.

## AUTHOR CONTRIBUTIONS

Yong Wang and Min Wang recorded the core data and drafted it. Zhao‐Qiong Zhu assisted in completing the manuscript and approved the final version. Rui Jiang designed the image; Guang‐Cai Li guided the completion of the revised manuscript.

## CONFLICT OF INTEREST STATEMENT

The authors declare no conflict of interest.

## ETHICS STATEMENT

This study was approved by the Guizhou Aerospace Hospital Medical Ethics Review Committee [2022]1‐049. The informed consent was signed by the patient.

## Data Availability

The data used and/or analyzed during the current study can be obtained from the corresponding authors upon reasonable request.
